# Crystal structure of heliorhodopsin 48C12

**DOI:** 10.1038/s41422-019-0266-0

**Published:** 2019-12-26

**Authors:** Yang Lu, X. Edward Zhou, Xiang Gao, Na Wang, Ruixue Xia, Zhenmei Xu, Yu Leng, Yuying Shi, Guangfu Wang, Karsten Melcher, H. Eric Xu, Yuanzheng He

**Affiliations:** 1 Laboratory of Receptor Structure and Signaling, HIT Center for Life Sciences, Harbin Institute of Technology, Harbin, Heilongjiang 150001 China; 2 Structural Biology Program, Van Andel Research Institute, Grand Rapids, MI 49503 USA; 3 Laboratory of Neuroscience, HIT Center for Life Sciences, Harbin Institute of Technology, Harbin, Heilongjiang 150001 China; 4 Center for Structure and Function of Drug Targets, Key Laboratory of Receptor Research, Shanghai Institute of Materia Medica, Chinese Academy of Sciences, Shanghai, 201203 China

**Keywords:** Nanocrystallography, Hormone receptors

Dear Editor,

Heliorhodopsins (HeRs) are a new class of Microbial rhodopsins (MRs), with a low homology (<15%) to all other MRs and a unique membrane topology in which their N-termini face the intracellular side and C-termini face the extracellular side.^1^ To explore the photoactivation mechanism of HeRs, we sought to solve the structure of HeR 48C12 (hereafter referred to as HeR). HeR protein was expressed in insect cells and purified by standard membrane purification methods (Supplementary information, Fig. S1a and Data S1). The purified HeR shows a monodispersed peak and exhibits a high thermostability (Tm = 74 °C, Supplementary information, Fig. S1b, c). Well-diffracting HeR protein crystals were grown in lipid cubic phase (Supplementary information, Fig. S1d, e), and the structure was solved at 2.7 Å resolution (Supplementary information, Data S1 and Table S1). The structure contains two HeR molecules in each asymmetric unit (designated as chains A and B) and the two molecules align very well with each other (RMSD = 0.361 Å, Supplementary information, Fig. S2a). We have successfully assigned almost the entire protein to the structure except 7 disordered residues in N/C-termini and 5 disordered residues (residues 94–97) in the intracellular loop 1. Despite the unique topology and low homology to other MRs, the structure of HeR shows a typical seven transmembrane helix bundle of MRs (Fig. 1a). The most striking feature of the HeR structure is the very long extracellular loop 1 (ECL1) that is mainly composed of two antiparallel β-strands, which has never been seen in other rhodopsins. An examination of surface electrostatics shows that the cytoplasmic side (N-terminus) is highly positively charged while the extracellular side is slightly negatively charged (Fig. 1c).

**Fig. 1:**
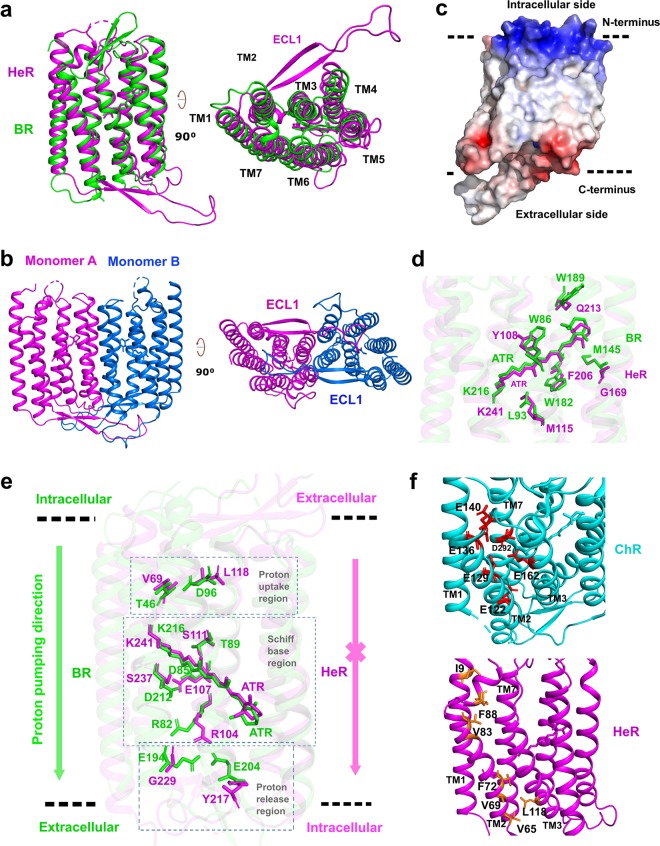
Structure of HeR 48C12. **a** The overall structure of HeR. For comparison, the HeR structure is superimposed onto the BR structure (1C3W). **b** The overall dimer structure of HeR. **c** The electrostatic surface view of HeR. Red and blue correspond to potentials of −5 kT *e*^−1^ and 5 kT *e*^−1^, respectively. **d** The ATR core of HeR and BR (1C3W). **e** Structural comparison of the proton pumping chain of BR (1C3W) with the corresponding region in HeR. The magenta cross indicates that the proton pumping path is blocked in HeR. **f** Structural comparison of HeR with channelrhodopsin C1C2 (3UG9) in the potential ‘channel’ region. The negatively charged residues distributed in the tunnel formed by TM1, 2, 3 and 7 of channelrhodopsin C1C2 are marked in red, while the hydrophobic residues localized in the counterpart region of HeR are marked in brown gold.

The structure also revealed that HeR forms a dimer (Fig. 1b). The dimer interface is mainly formed by the extensive hydrophobic interaction between two perpendicular interfaces: 1) the interaction between the ECL1 of monomer A and ECL2/3 in monomer B at the extracellular side; 2) the interaction between transmembrane helices TM4 and TM5 of the two monomers (Supplementary information, Fig. S3a, b). The overall dimer interface is 2260 Å^2^ as calculated by ccp4 program PISA,^2^ which is the most extensive dimerization interface seen in MRs. We investigated the dimer formation in physiological environment by confocal microscopy. The eGFP- and mRFP-fused HeRs were co-transfected into AD293 cells. The confocal images show that the eGFP/mRFP-labeled HeRs are tightly associated with each other on the membrane (Supplementary information, Fig. S4a). Next, we performed deletion/mutation experiments to study the role of dimerization in HeR’s function. We first analyzed the F144A/Y151S mutation, which interferes with the hydrophobic interaction between TM4 and TM5 of the two monomers. The F144A/Y151S mutation caused a dramatic decrease of the protein yield and a significant loss of purple color (Supplementary information, Fig. S4b). We then deleted the long ECL1 [Δ(38–63)] that has extensive interaction with ECL2/3 in the dimer formation. Deletion of ECL1 caused a complete loss of all-*trans*-retinal (ATR) binding as characterized by the complete loss of purple color, and a predominant aggregation with a small oligomer shoulder peak in the size column profiling. A combination of the ECL1 deletion with the F144A/Y151S mutation caused a complete aggregation and complete loss of purple color of the protein, suggesting that dimerization is needed for the function of HeR.

Retinal is the ‘core’ of rhodopsin and is generally in all-*trans* configuration in the resting state. We then looked at the ATR positon in the hydrophobic core of the seven helix bundle. The ß-ionone ring and the long polyethylene chain of ATR are clearly defined by the electron density map (Supplementary information, Fig. S2b). Compared to the classic bacterial rhodopsin (BR),^3,4^ the hydrophobic core architecture for ATR binding is well conserved in HeR; in particular, the π-electron system formed by two aromatic residues (W182/W86 for BR, F206/Y108 for HeR) at both sides of ATR, is critical for stabilizing the protonated polyethylene chain of ATR (Fig. 1d). A previous study of HeR showed that HeR does not contain any pump or channel activity and suggested that protons are never being released from this protein.^1^ We therefore sought to understand these features at a structural level.

First, we performed pump activity assay to confirm the lack of pump activity of HeR. Similar to a previous study,^1^ illumination of HeR-expressing *E.  coli* cells did not change the pH of the external medium while green-absorbing proteorhodopsin-expressing cells, showed a quick drop of pH (Supplementary information, Fig. S5). The classic proton pumping chain of BR contains three parts: the proton uptake region, the Schiff base region and the proton release region.^5^ The proton uptake region is close to the cytoplasmic side, with two key residues, T46 and D96, that mediate the proton uptake. In the Schiff base region, the proton transferring is mediated by a series of polar and hydrogen network interactions (including water molecules), with key residues T89, D85, D212 and R82. The proton release region is close to the extracellular side, and contains two key residues, E204 and E194. Compared to BR, several key residues are missing in the HeR structure. For example, in the proton uptake region, the key proton donor D96 of BR is replaced by L118 in HeR. In the Schiff base region, the key residue D212 is changed to S237 in HeR; and most dramatically the key residues for proton release, E204 and E194, are missing in HeR (Fig. 1e). Together, the absence of these key residues likely explain the lack of pumping activity of HeR.

Next, we explored whether HeR could function as a channel. A brief illumination of ChRmine^6^ induced a rapid depolarizing current (~1–2 nA). In contrast, illumination of HeR did not induce any measurable current across the membrane of AD293 cells (Supplementary information, Fig. S6). Unlike pumping, which is mainly mediated through the ‘tunnel’ formed by TM3–7 with ATR in the center, the channel in channelrhodopsins is mainly formed by TM1, 2, 3, and 7 and does not enclose ATR. Generally, charged residues lining the channel pore define the selectivity of the channel. For instance, in chanelrhodopsin C1C2, E122, E129 and E136 from TM2, E162 from TM3 and D292 from TM7 define the ion conductance and cation selectivity^7^ (Fig. 1f, top panel). In contrast, the ‘tunnel’ formed by TM1, 2, 3 and 7 of HeR is filled with hydrophobic residues, e.g., F88, V83, F72, V69 and V65 of TM2, L118 of TM3, and I9 of TM1 (Fig. 1f, lower panel), which occlude ions from passing through this tunnel. In addition, we observed hydrophobic residues on both the extracellular side and intracellular side of HeR that block the entry of the ‘tunnel’ (Supplementary information, Fig. S7).

Finally, we sought to understand the long photocycle and proton release of HeR based on the structural information. The earlier study proposed H23 and H80 as proton-accepting groups and showed that the proton has never been released from the protein.^1^ Our structure shows that H23 and H80 are isolated from detectable polar interaction to the proton transferring chain centered at E107 (Supplementary information, Fig. S8a). Therefore, the proton cannot be directly passed on to H23 or H80. Although there is a hydrogen bond between S76 and E107, S76 itself cannot serve as a proton acceptor; therefore, the proton is stuck at the core region. Our structure did not reveal water molecules in this region. Even in the case that water molecules exist in this region, both ends of the HeR TM1, 2, 3, 7 tunnel (where H23/H80 localize) are filled with hydrophobic residues (Fig. 1f, lower panel). Therefore, the photon cannot escape from the tunnel, and eventually return back to the Schiff base, which may explain the long photocycle of HeR. To validate our hypothesis, we mutated the key residues (H23A, H23S, H80A and E107A) of the proton transferring chain of this region. The H23A, H23S and H80A mutations show the same single λ_max_ of 550 nm as wild type under most conditions (Supplementary information, Fig. S8b, c), indicating that these residues do not act as proton acceptors. On the other hand, purified HeR E107A showed a major 410 nm and a minor 550 nm peak under the purification condition (pH 6.8, 300 mM NaCl). With the decrease of salt concentration, the 550 nm peak of E107A decreased and eventually disappeared at 75 mM salt concentration (Supplementary information, Fig. S8b). Moreover, E107A at pH 4.0 showed a single λ_max_ of 550 nm that decreased with gradually increased pH, while the of 410 nm peak increased and eventually formed a single λ_max_ 410 nm at pH 11 (Supplementary information, Fig. S8c, d). These data suggested that the acidic E107 is the main counterion for the protonated Schiff Base. Under the high salt condition, the negatively charged anion (Cl^–^) may take the place of E107A to counteract the protonated Schiff base, maintaining the main 550 nm peak; under the low salt condition, since no negatively charged anion is available, the Schiff base is deprotonated, which explains the appearance of the 410 nm peak. At the low pH 4.0, the Schiff base remains protonated so that λ_max_ of 550 nm is maintained; with increase of pH, the Schiff base starts to be deprotonated so that the 410 nm peak appears (Supplementary information, Fig. S8c). Taken together, these data suggest that E107 plays an essential role in proton transferring, while H23 and H80 are not essential.

While we were preparing this manuscript on HeR 48C12, we noted that a structure of a HeR homolog, *Thermoplasmatales* archaeon HeR (*Ta*HeR), was published online in *Nature*.^8^ The structure is very similar to our HeR 48C12 structure with a RMSD of 0.693 Å (Supplementary information, Fig. S9a). Both structures revealed the unique long ECL1, a distinguished dimer interface and core architectures that hold ATR in positions. However, sequence variations between these two HeR proteins, particularly those lining the ATR-binding pocket, may indicate functional differences between these two proteins (Supplementary information, Fig. S9b). Overall, we have reported the novel structure of HeR 48C12 and mechanistically addressed the key physiological properties of HeR, such as pump/channel activity and the long photocycle.

The atomic coordinates and structure factors were deposited in the Protein Data Bank under accession number 6UH3.

## Supplementary information

Supplementary information
