# Supplementary material for: Crystal structure of heliorhodopsin 48C12
Source: Cell Res. 2019 Dec 26;30(1):88–90. doi: 10.1038/s41422-019-0266-0 (PMC6951262; doi:10.1038/s41422-019-0266-0)
Supplement: Supplementary file 1 — Supplementary information [file 41422_2019_266_MOESM1_ESM.pdf]

**Supplementary Information**

# Crystal structure of heliorhodopsin 48C12

Yang Lu[1]*, X. Edward Zhou[2]*, Xiang Gao[2], Na Wang[1], Ruixue Xia[1], Zhenmei Xu[1], Yu Leng[1], Yuying Shi[3], Guangfu Wang[3], Karsten Melcher[2], H. Eric Xu[2,4] and Yuanzheng He[1#]

[1] Laboratory of Receptor Structure and Signaling, HIT Center for Life Sciences, Harbin Institute of Technology, Harbin 150001, China

[2] Structural Biology Program, Van Andel Research Institute, Grand Rapids, MI 49503, USA

[3] Laboratory of Neuroscience, HIT Center for Life Sciences, Harbin Institute of Technology, Harbin 150001, China

[4] Center for Structure and Function of Drug Targets, Key Laboratory of Receptor Research, Shanghai Institute of Materia Medica, Chinese Academy of Sciences, Shanghai 201203, China

Correspondence: Yuanzheng He

Email: ajian.he@hit.edu.cn

## Materials and Methods

### Protein expression and purification

Wild type (wt) HeR gene containing an N-terminal histidine-tag and MBP-tag followed by the TEV protease cleavage site was cloned into pFastBac vector. This construct was expressed in *Spodoptera frugiperda* (Sf9) cells using the baculovirus infection system. The collected cells were disrupted by sonication in buffer containing 50 mM Tris-HCl (pH 6.8), 300 mM NaCl and protease inhibitor cocktail (Roche). The crude membrane fraction was centrifuged at 208,500 g for 50 min. The membrane fraction was solubilized in buffer containing 50 mM Tris-HCl (pH 6.8), 300 mM NaCl, 0.5% (w/v) n-dodecyl-β-D-maltoside (DDM; Biosynth) and 0.1% (w/v) cholesteryl hemisuccinate (CHS; Anatrace), for 2 h at 4 ℃. The sample was centrifuged at 208,500 g for 50 min to remove debris, and the supernatant was incubated with Amylose Resin (NEB) at 4 ℃ overnight. Then, the resin was washed with 15 column volumes of 50 mM Tris-HCl (pH 6.8), 300 mM NaCl, 0.02% DDM (w/v) and 0.004% CHS (w/v). The receptor was eluted with 4 column volumes of 50 mM Tris-HCl (pH 6.8), 300 mM NaCl, 0.02% DDM (w/v), 0.004% (w/v) CHS and 10 mM D-(+)-Maltose monohydrate (Sigma-Aldrich). The eluted protein was concentrated to 1 ml with a 100-kDa cutoff concentrator (Millipore) and TEV protease (in-house prepared) was added for 4 h at room temperature. Cleaved HeR protein was further purified by reloading over Ni-sepharose resin (Qiagen). The flow through was collected and purified by size-exclusion chromatography using a Superdex 200 10/300 column (GE Healthcare).

### Crystallization of HeR

The protein was concentrated to about 15 mg ml$^{-1}$ with a 100-kDa cutoff concentrator and reconstituted into the lipidic cubic phase (LCP) by mixing with monoolein at a protein/lipid ratio of 1:1.5. The protein: lipid mixture was delivered through an LCP dispensing robot (Gryphon; Art

Robbins Instruments) in 50 nl drops to 96-well glass sandwich plates and overlaid en-bloc with 800 nl precipitant solution. Crystals for data collection were grown in 50 mM sodium citrate tribasic dihydrate pH 5.4, 350 mM potassium citrate tribasic monohydrate, 37% PEG400, 0.5% w/v DDM. Crystals reached full size within 7-10 days at 20 ℃ and were picked from a sponge-like mesophase and flash-frozen in liquid nitrogen without additional cryoprotectant.

**Confocal microscopy imaging**

AD293 cells were split into 12 well plates with a 15 mm cover slid in the center of the well at 40,000 cells per well. 100 ng pcDNA3-eGFP-HeR were co-transfected with 100 ng pcDNA3-mRFP-HeR into AD293 cells by X-tremeGENE HP (Roche) according to the manufacturer's instruction. One day after transfection, the cover slid was picked up and mounted into the Carl Zeiss Laser scanning confocal microscope (LSM880 with fast airyscan). The confocal images were captured and analyzed by software ZEN 2.6 (blue edition).

**Pump-activity assay of rhodopsins**

*E. coli* cells expressing rhodopsins were collected by centrifugation at 3000 rpm for 20 min and were washed three times with an unbuffered salt solution (100 mM NaCl and 10 mM $MgCl_2$). After that, the cells were resuspended in 100 mM NaCl solution and adjusted to an OD600 of 15. The cells were illuminated for 3 min using a halogen lamp and the light-induced pH changes were monitored with a pH meter (METLIER TOLEDO). Measurements were repeated under the same conditions after the addition of 30 μM CCCP.

**Electrophysiology**

Patch-clamp recordings of AD293 cells were performed as previously described.[1] Briefly, whole-cell recordings were obtained from a eGFP-HeR or ChRmine-eYFP [2] expressing cell under visual guidance with fluorescence and transmitted light illumination using an Axopatch-200B amplifier (Molecular Devices). Bath solution (30 ± 1.5°C) contained 119 mM NaCl, 2.5 mM KCl, 2 mM

$CaCl_2$, 1 mM $MgCl_2$, 26 mM $NaHCO_3$, 1 mM $NaH_2PO_4$, 25 mM glucose (pH 7.4) gassed with 5% $CO_2$/95% $O_2$. Patch recording pipettes (4-8 MΩ) contained 120 mM potassium gluconate, 10 mM HEPES, 4 mM KCl, 4 mM MgATP, 0.3 mM $Na_3$GTP, 10 mM sodium phosphocreatine and 0.5% biocytin (pH 7.25). A motorized micromanipulator (Junior Multpatch, Lugis & Neumann) was used to steer the pipette electrode to the target cell. Green light for optical stimulation was provided by the excitation system of a fluorescence microscope (BX51WI, Olympus). In brief, light from a 100 W Mercury lamp (U-LH100HG) was attenuated by two 6% and 25% neutral density filters (U-25ND6 and U-25ND25), filtered by a fluorescence filter cube with a 530-550 band-pass filter (U-MWIG3), and then delivered to the cells through a 40X objective (LUMPLFLN40XW). The shutter of the illumination source was controlled manually. The timing of the stimulation was determined by monitoring the fluorescence of 48C12-eGFP- or ChRmine-eYFP-expressing cells via a digital CMOS camera (ORCA-FLASH4.0, Hamamatsu Photonics) working in external trigger mode. Electrophysiological data acquisition and camera triggering were operated by a custom-written Igor Pro 6 program (WaveMetrics)[1, 3] with modifications for controlling LIH 8+8 data acquisition interface (HEKA Instruments).

**Full-wavelength absorption assay**

Wild type or mutant HeR with a 6xHis tag at their N-termini were expressed in BL21(DE3) and induced and purified as before. The proteins were concentrated to about 5 mg/ml and the full-wavelength (200 – 900 nm) absorption were measured by IMPLEN N60 TOUCH spectrometer.

**Data collection and structure determination**

X-ray diffraction data were mainly collected at Advanced Photon Source (APS, Chicago, USA) at beam line 21-ID-D using a wavelength of 1.0 Å. Additional data were collected at SPring-8

BL41XU at Sayo, Japan. The HeR structure was solved by molecular replacement with phenix.mr_rosetta,[4] using the Acetabularia Rhodopsin II model (PDB code: 3AM6).[5] The model was then refined by phenix.refine [6] and manually adjusted by COOT.[7]

## References


1 Wang G, Zhang P, Mendu SK *et al.* Revaluation of magnetic properties of Magneto. *Nat Neurosci* 2019.

2 Marshel JH, Kim YS, Machado TA *et al.* Cortical layer-specific critical dynamics triggering perception. *Science* 2019; **365**.

3 Wang G, Wyskiel DR, Yang W *et al.* An optogenetics- and imaging-assisted simultaneous multiple patch-clamp recording system for decoding complex neural circuits. *Nature protocols* 2015; **10**:397-412.

4 DiMaio F, Terwilliger TC, Read RJ *et al.* Improved molecular replacement by density- and energy-guided protein structure optimization. *Nature* 2011; **473**:540-543.

5 Wada T, Shimono K, Kikukawa T *et al.* Crystal structure of the eukaryotic light-driven proton-pumping rhodopsin, Acetabularia rhodopsin II, from marine alga. *J Mol Biol* 2011; **411**:986-998.

6 Afonine PV, Grosse-Kunstleve RW, Echols N *et al.* Towards automated crystallographic structure refinement with phenix.refine. *Acta Crystallogr D Biol Crystallogr* 2012; **68**:352-367.

7 Emsley P, Lohkamp B, Scott WG, Cowtan K. Features and development of Coot. *Acta Crystallogr D Biol Crystallogr* 2010; **66**:486-501.

**Supplementary information, Table S1. Data collection and structure refinement statistics**

| | Heliorhodopsin 48C12 |
|---|---|
| **Data collection** | |
| Space group | P21 |
| Resolution range, Å | 50-2.7(2.83-2.70)* |
| Cell parameters (a, b, c; α, β, γ) Å, ° | 49.4, 102.1, 56.8; 90, 94,90 |
| Total/Unique reflections | 43713/14449 |
| Completeness, % | 93.3 (91.0) |
| Mean I/σ | 4.8 (2.0) |
| Multiplicity | 3.2 (3.1) |
| Rmerge | 0.23 (0.98) |
| CC1/2 | 0.94 (0.31) |
| **Refinement** | |
| Resolution, Å | 49-2.6(2.84-2.70)* |
| No. reflections | 11595 (2709) |
| No. protein residues | 491 |
| No. solvent/lipid molecules | 64 |
| No. of ligands | 2 |
| No. non-H atoms | 4191 |
| Rcryst % | 26.6(32.7) |
| Rfree % | 28.0(34.8) |
| rmsd bonds, Å | 0.006 |
| rmsd angles, ° | 1.115 |
| Wilson B factor, Å2 | 30.0 |
| B factor of protein, Å2 | 35.8 |
| B factor of ligand, Å2 | 46.7 |
| Ramachandran favored/outlier % | 96.5/0.00 |
| Clash score | 5.61 |
| Molprobity score | 1.54 |

*Values in parentheses are for the highest resolution shell.

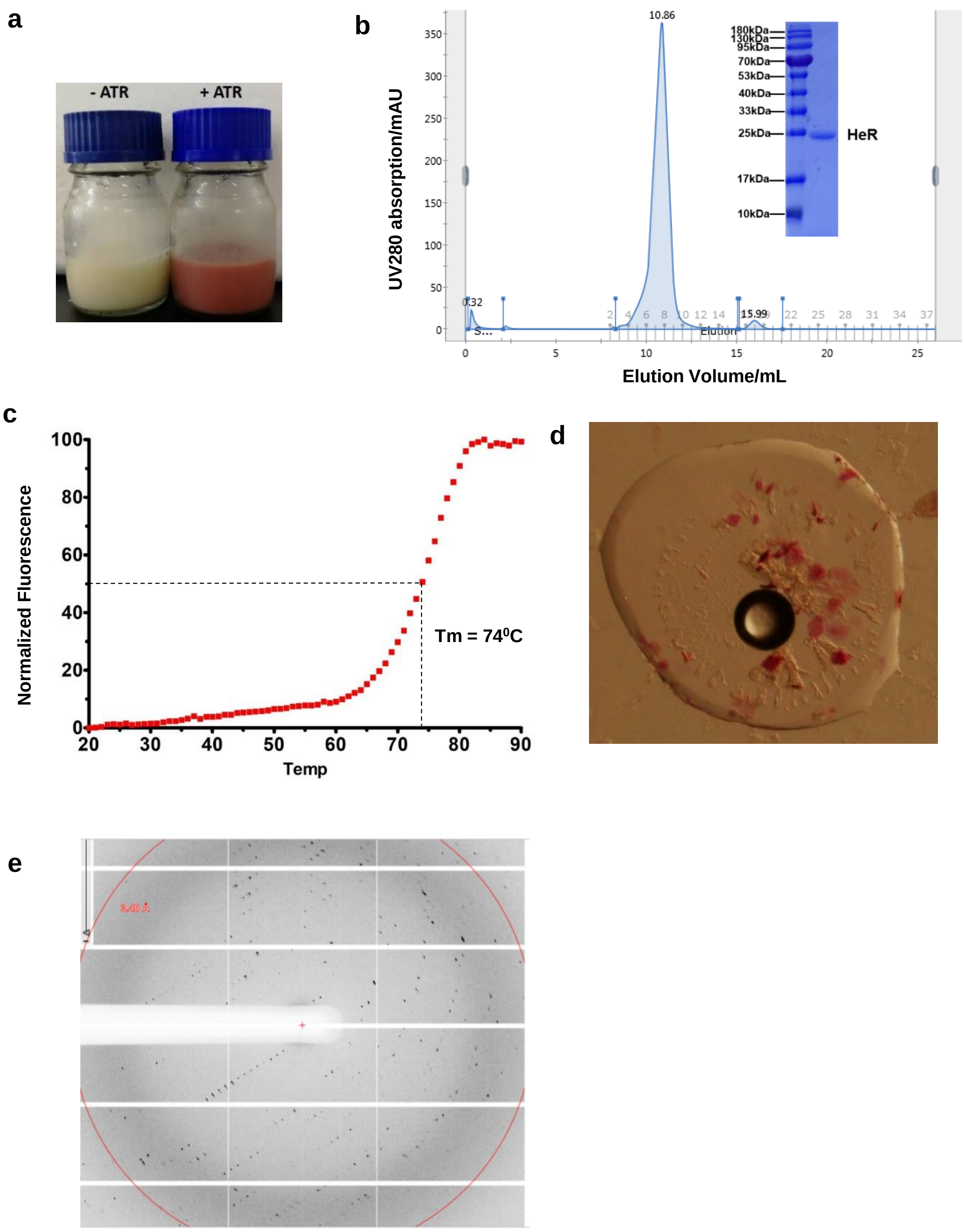


**Supplementary information, Fig. S1** Protein expression, purification and crystallization of HeR. **a** Representative color change of HeR-expressing cells when ATR is added to the cell culture. **b** Size profiling of HeR. **c** Thermostability shift assay of HeR. **d** HeR crystals grown in LCP. **e** X-ray diffraction pattern of HeR crystal.

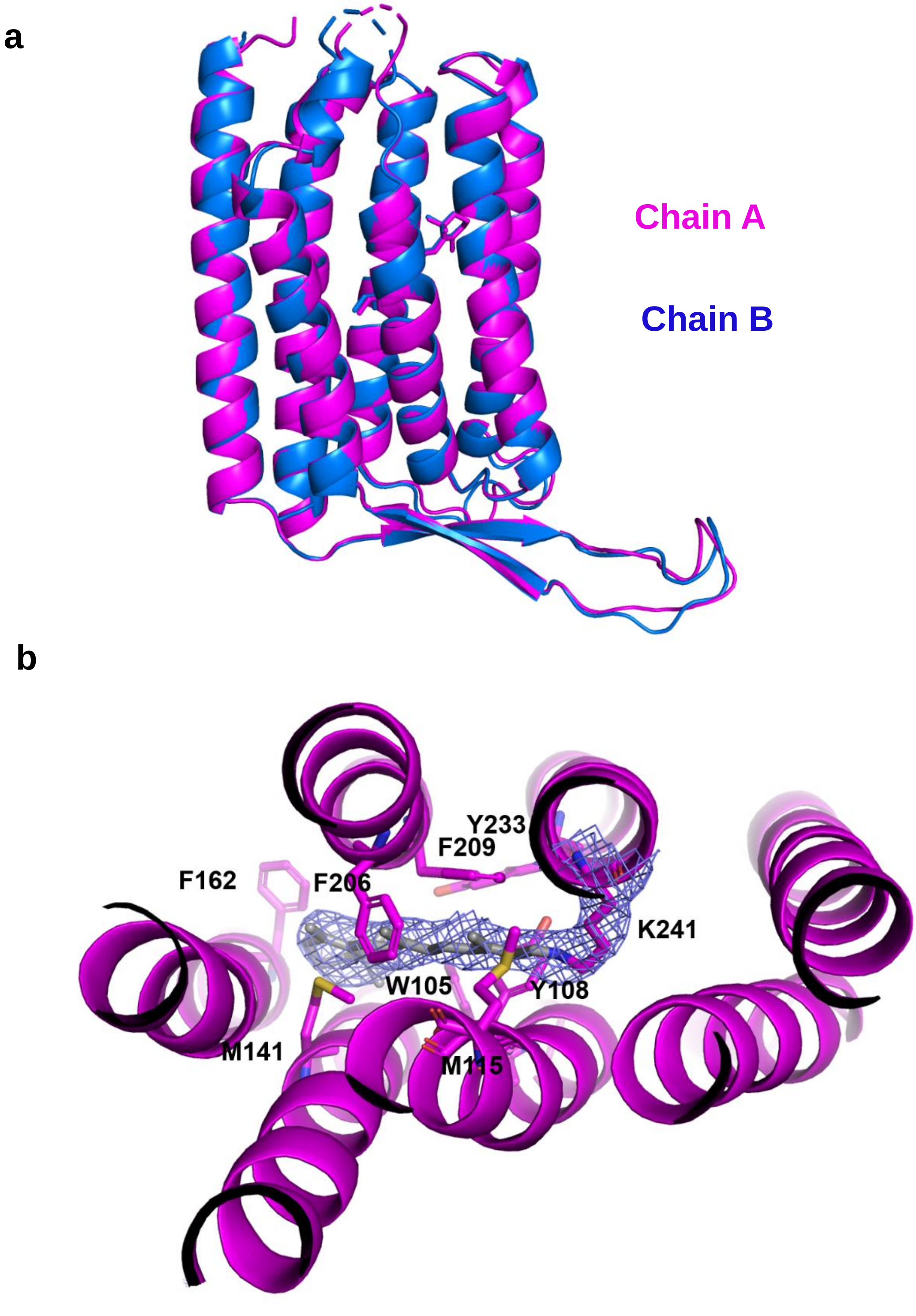


**Supplementary information, Fig. S2** Structural feature of HeR. **a** Superimposition of chain A with chain B of the asymmetry unit. **b** ATR density map and surrounding key residues. The purple mesh show the electron density maps (2*F*obs – *F*calc) of ATR at contour level, 1σ.

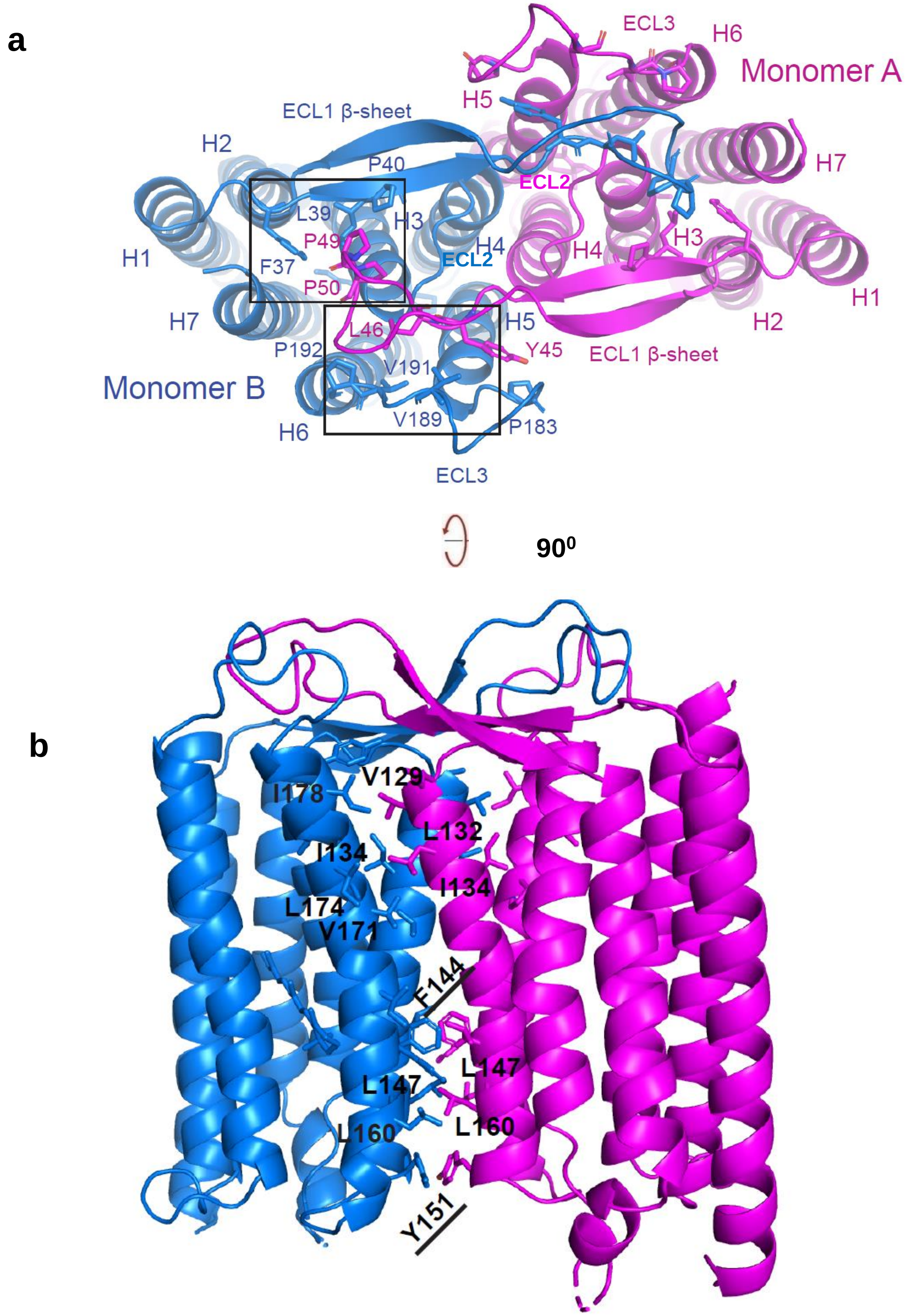


**Supplementary information, Fig. S3** The detail of HeR dimer interface. **a** The interface between ECL1 of monomer A with ECL2 and ECL3 in monomer B. **b** The hydrophobic interface between H4 and H5 of the two monomers.

a

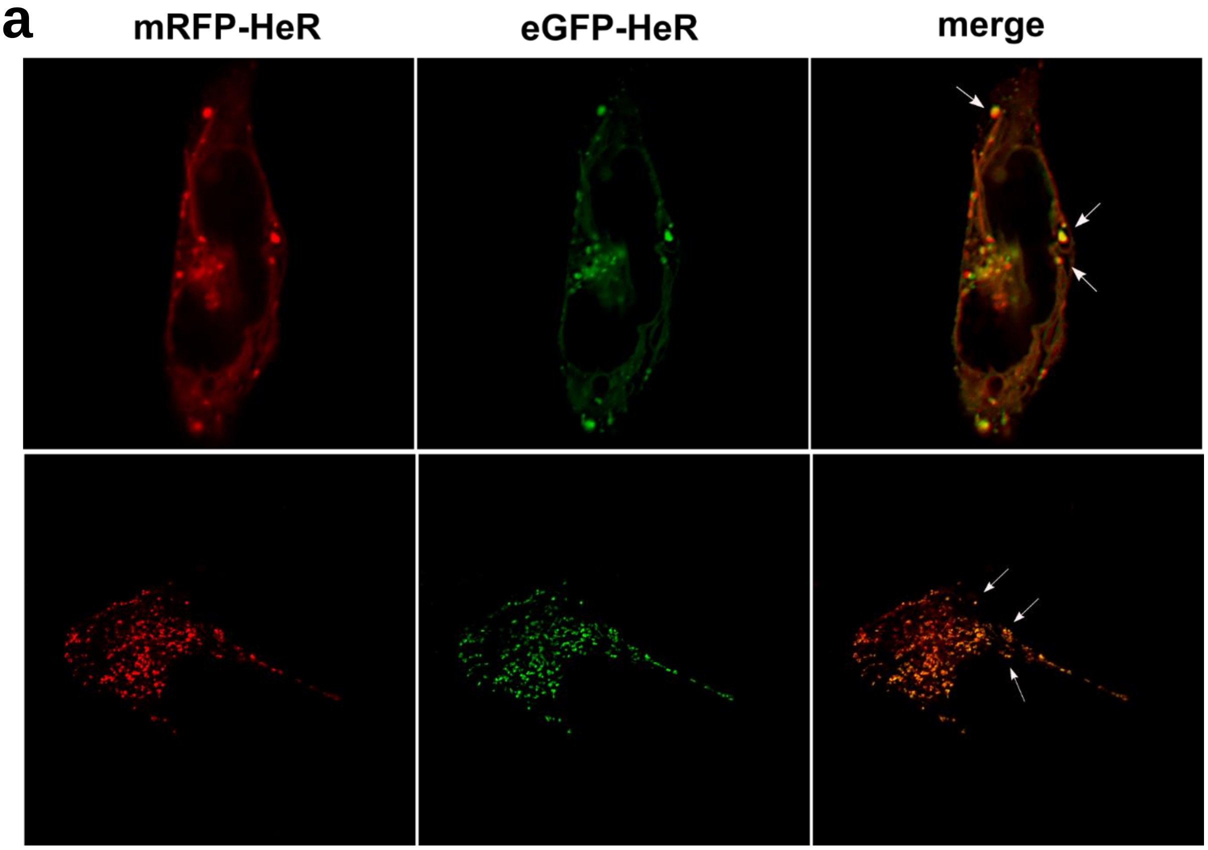


b

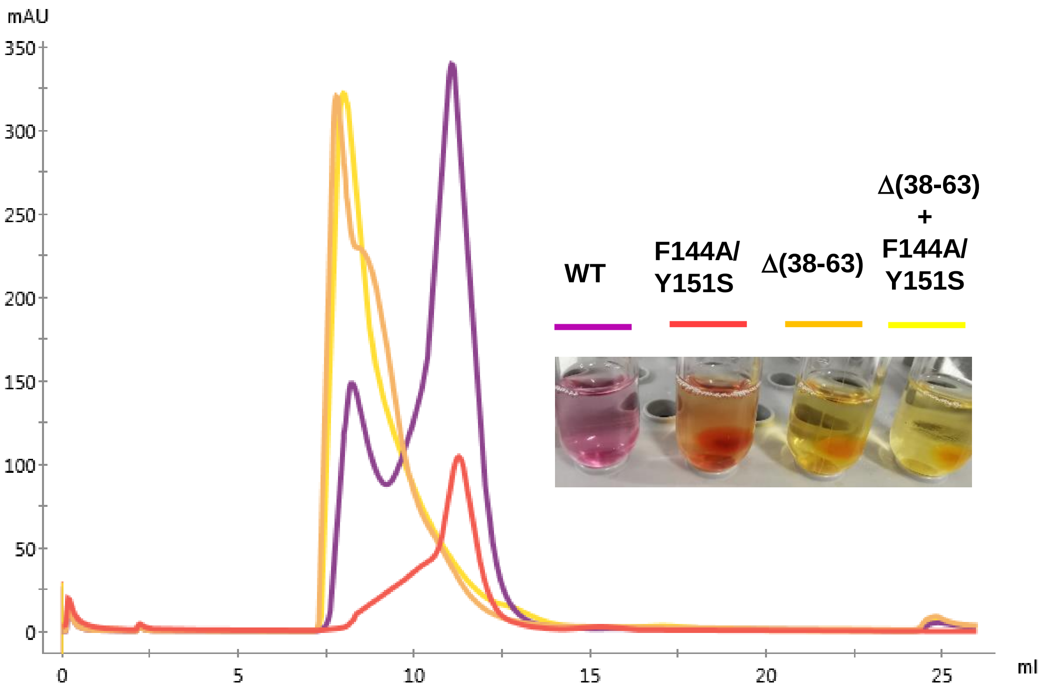


**Supplementary information, Fig. S4** HeR dimerization study. **a** Confocal images of the colocalization of mRFP-HeR with eGFP-HeR in AD293 cells. The white arrows in the merge panel are representative colocalization spots. **b** Size column profiling of HeR dimerization mutations. The color in the small tube indicates the solubilized membrane protein color. The aggregation peak is near 7.5 ml and the dimer peak at ~12.0 ml.

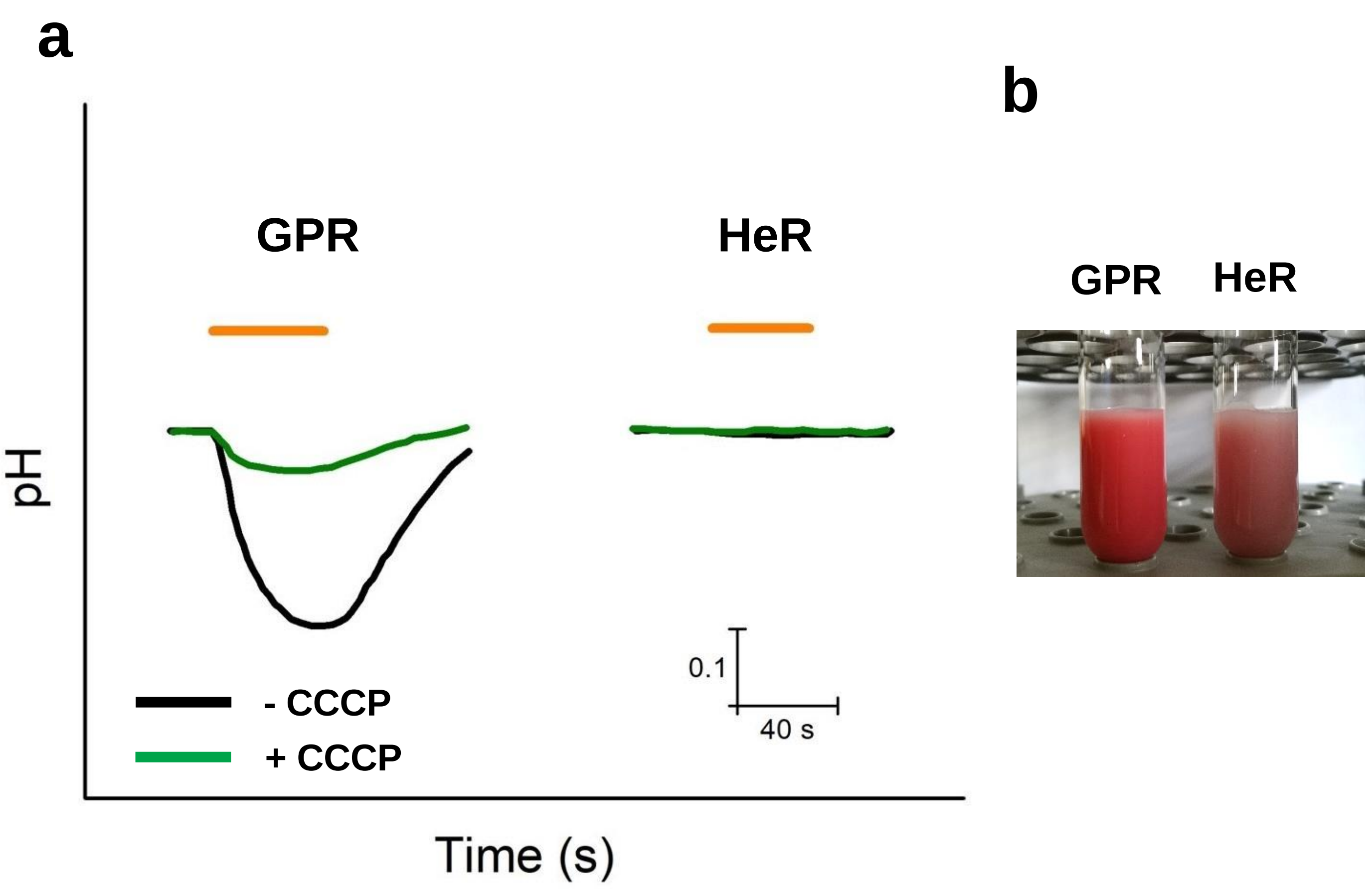


**Supplementary information, Fig. S5** Pump-activity of HeR. **a** The pump-activity of HeR and GPR. The orange line indicated the light illumination period. **b** Color of rhodopsin expressing cells used in the pump-activity assay.

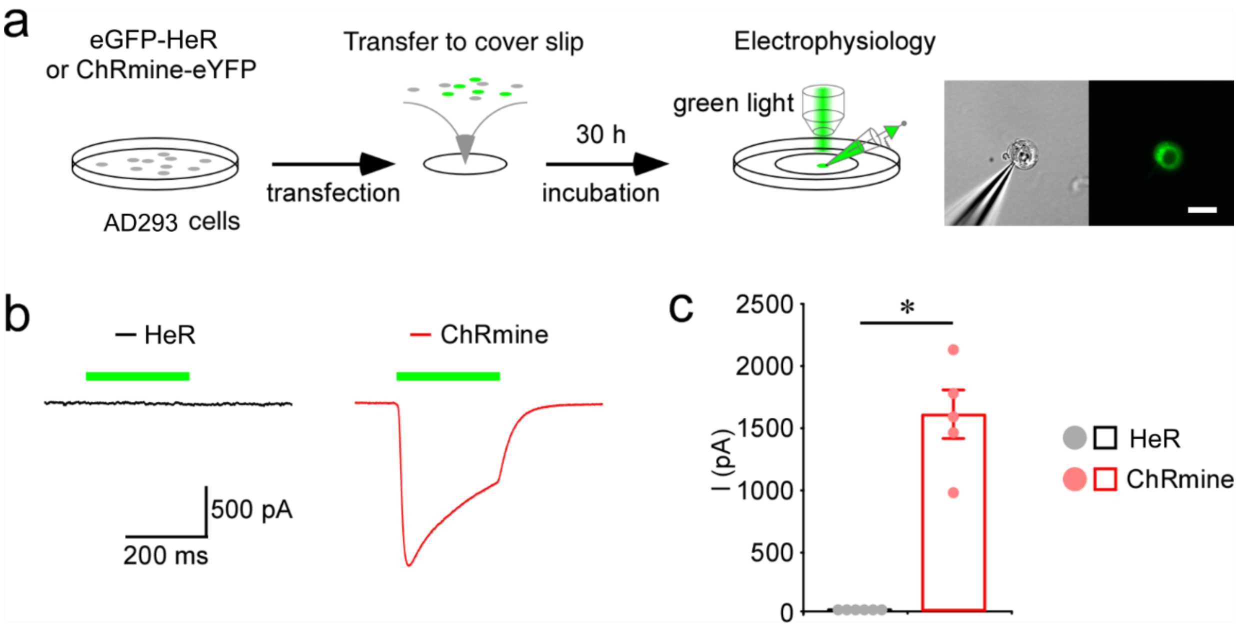


**Supplementary information, Fig. S6** Optical stimulation-induced inward currents in ChRmine-expressing, but not HeR-expressing, AD293 cells. **a** Schematic drawing outlining the design of *in vitro* transfection, optical stimulation and electrophysiological recording in HeR- and ChRmine-expressing cultured AD293 cells. The right images show whole-cell recording from a ChRmine-eYFP expressing cell under transmitted light (left) and fluorescence microscopy with GFP filter (right). Scale bar equals 20 µm. **b** Current recordings from a HeR expressing cell (left), and a ChRmine expressing cell (right) during optical stimulation with 540 nm green light. The membrane potential of AD293 cells was held at -60 mV. The green bars indicate the time when the green light was present. **c** Values of peak currents of HeR-expressing and ChRmine-expressing AD293 cells during optical stimulation (HeR: 1.2±0.3 pA, $n$=6; ChRmine: 1574.9±189.6 pA, $n$=5; $Z$=-2.647, $p$<0.01). Solid circles indicate peak currents of each recorded cell. Asterisk indicates $p$<0.01 (Wilcoxon test).

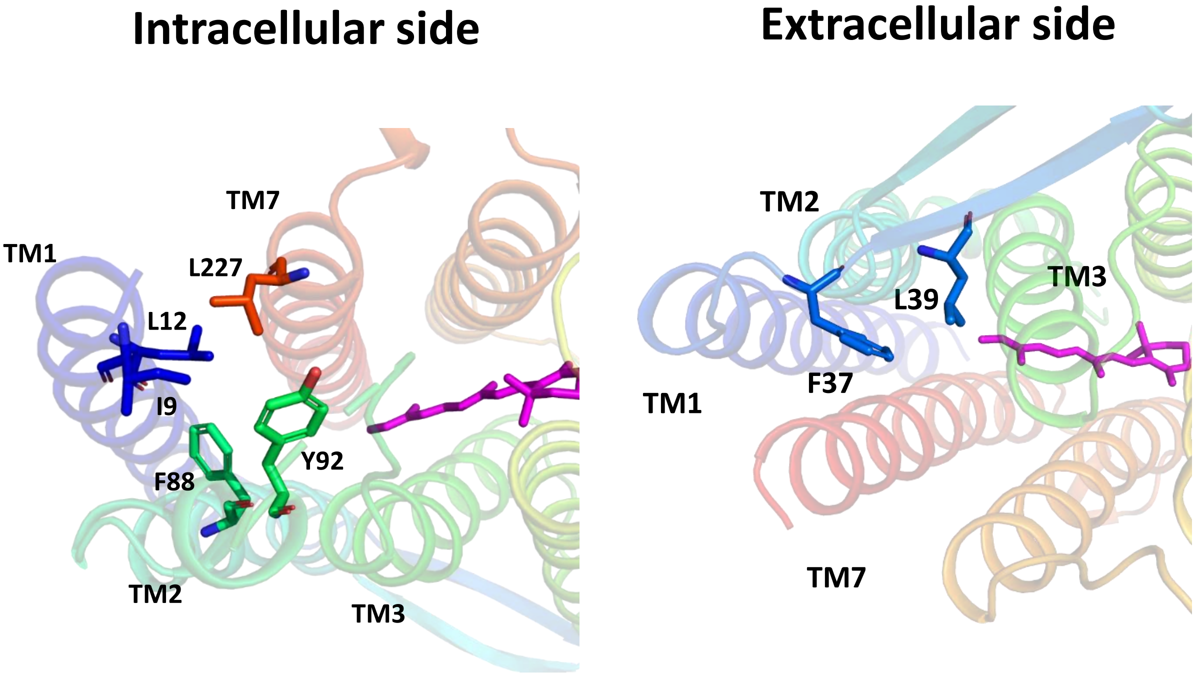


**Supplementary information, Fig. S7** Hydrophobic residues block the ‘tunnel’ entry of HeR on both extracellular and intracellular sides.

**a**

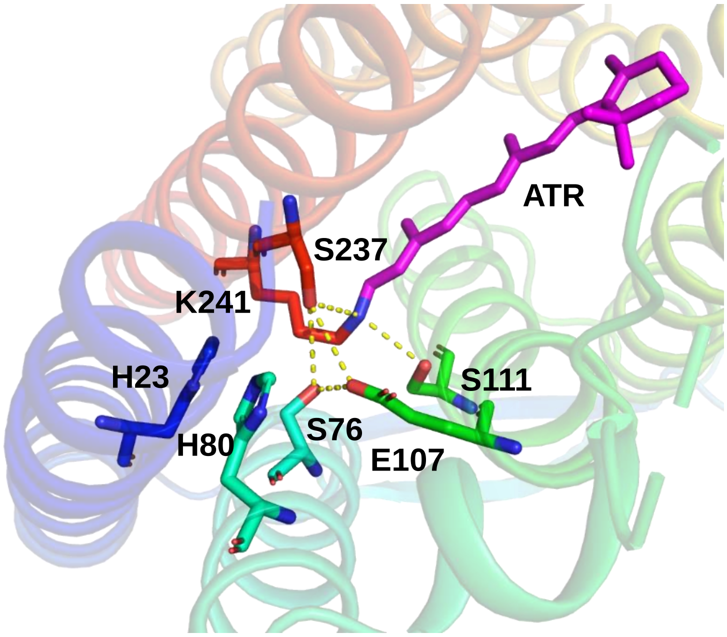


**b**

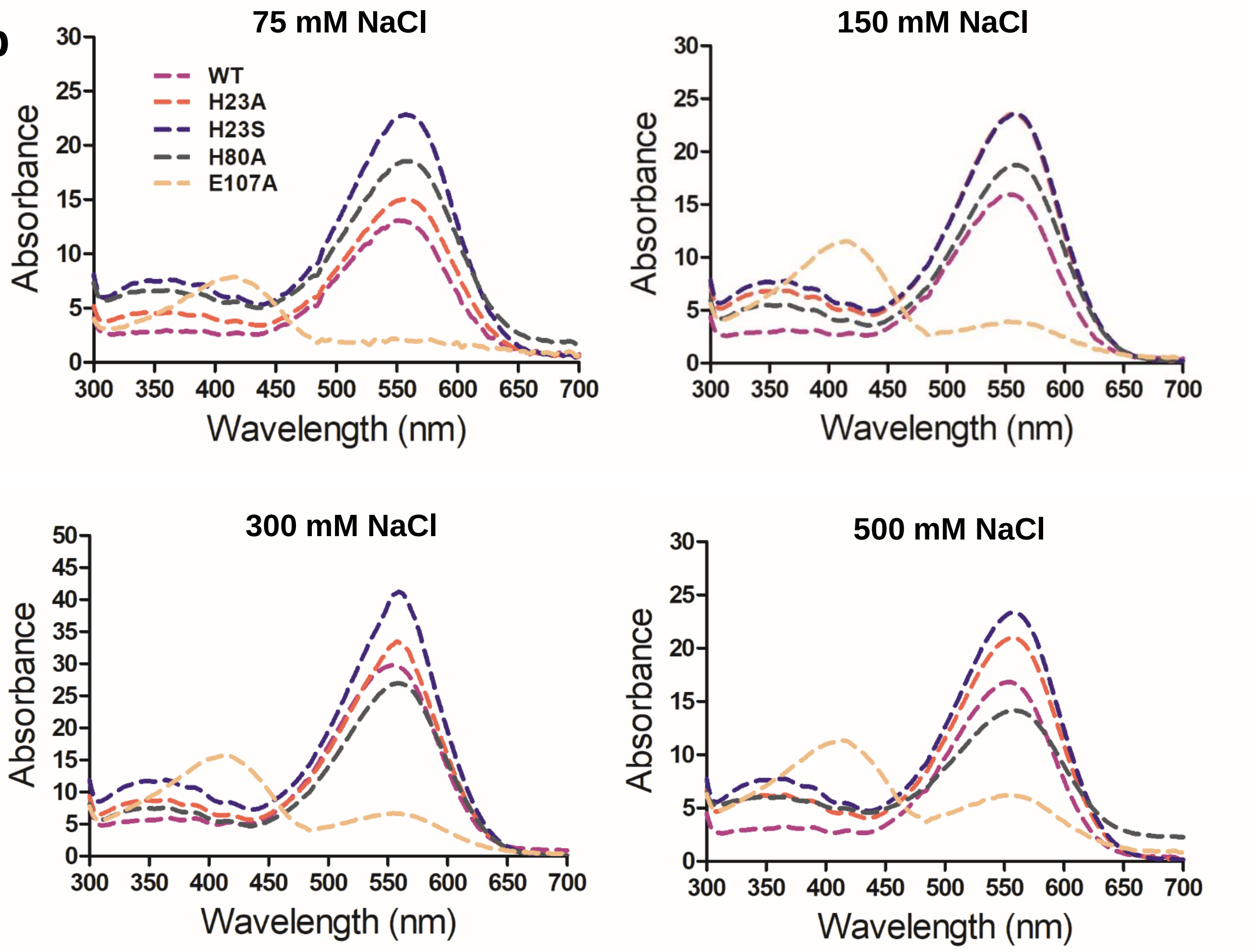


**Supplementary information, Fig. S8**

c

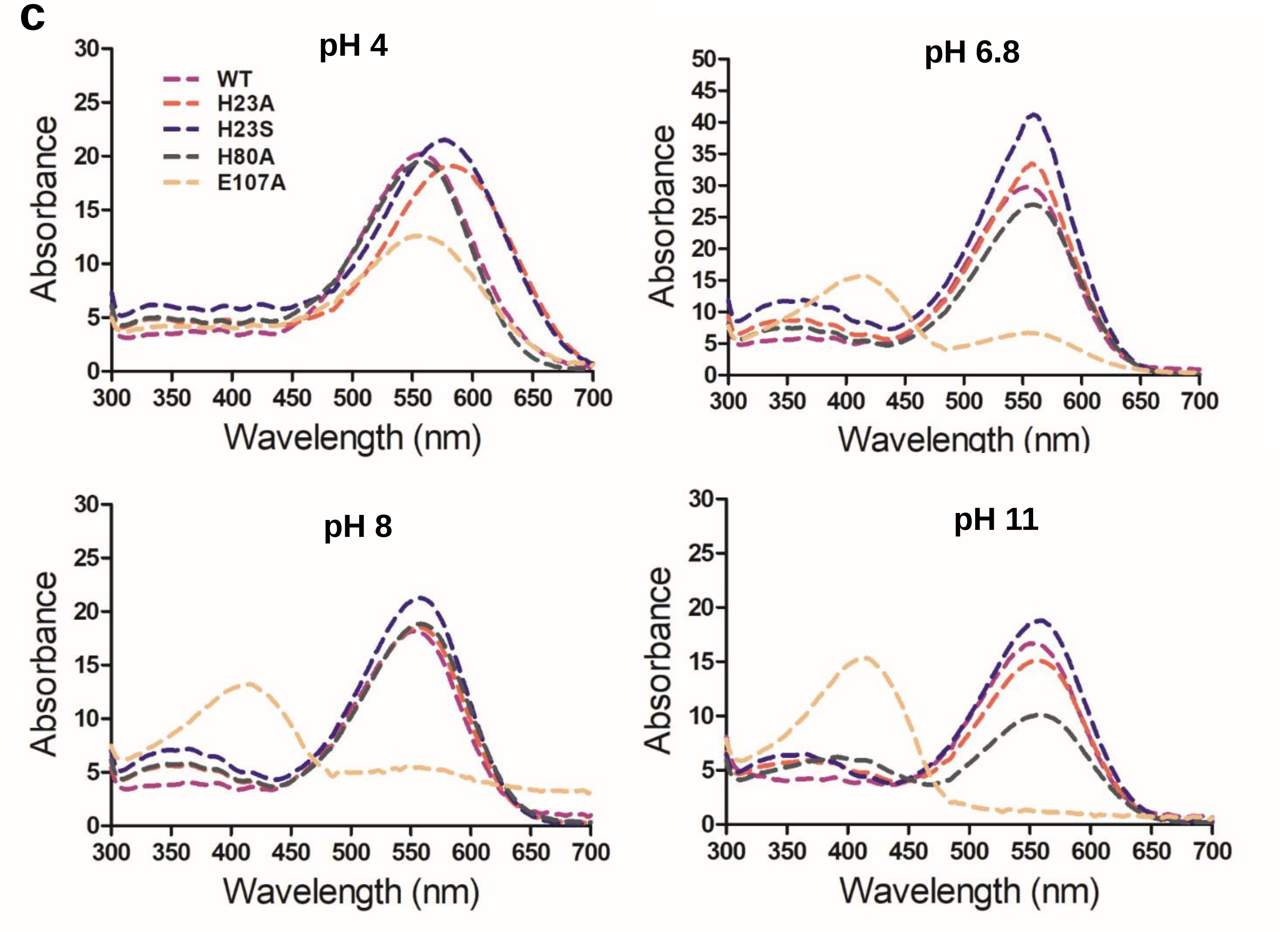


d

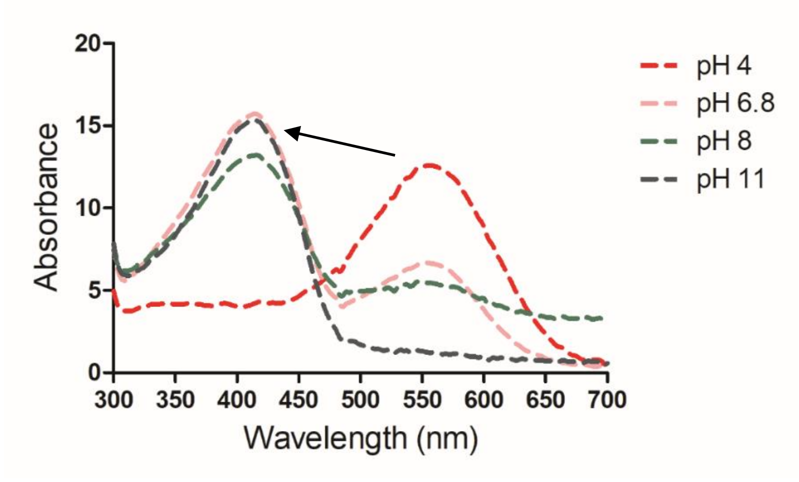


**Supplementary information, Fig. S8** Mechanistic insight into the proton transferring chain of HeR. **a** The polar interaction of the proton transferring center in HeR. H23 is isolated from any polar or charge interaction while E107 is in the proton transferring center. **b** The effect of salt concentration on the wavelength absorption of HeR mutations. **c** The effect of pH on the wavelength absorption of HeR mutations at salt concentration of 150 mM NaCl. **d** The effect of pH on the wavelength absorption of the E107A mutation of HeR at salt concentration of 150 mM NaCl. The solid arrow shows the major peak shift of E107A from 550 nm to 410 nm with the pH change from 4 to 11.

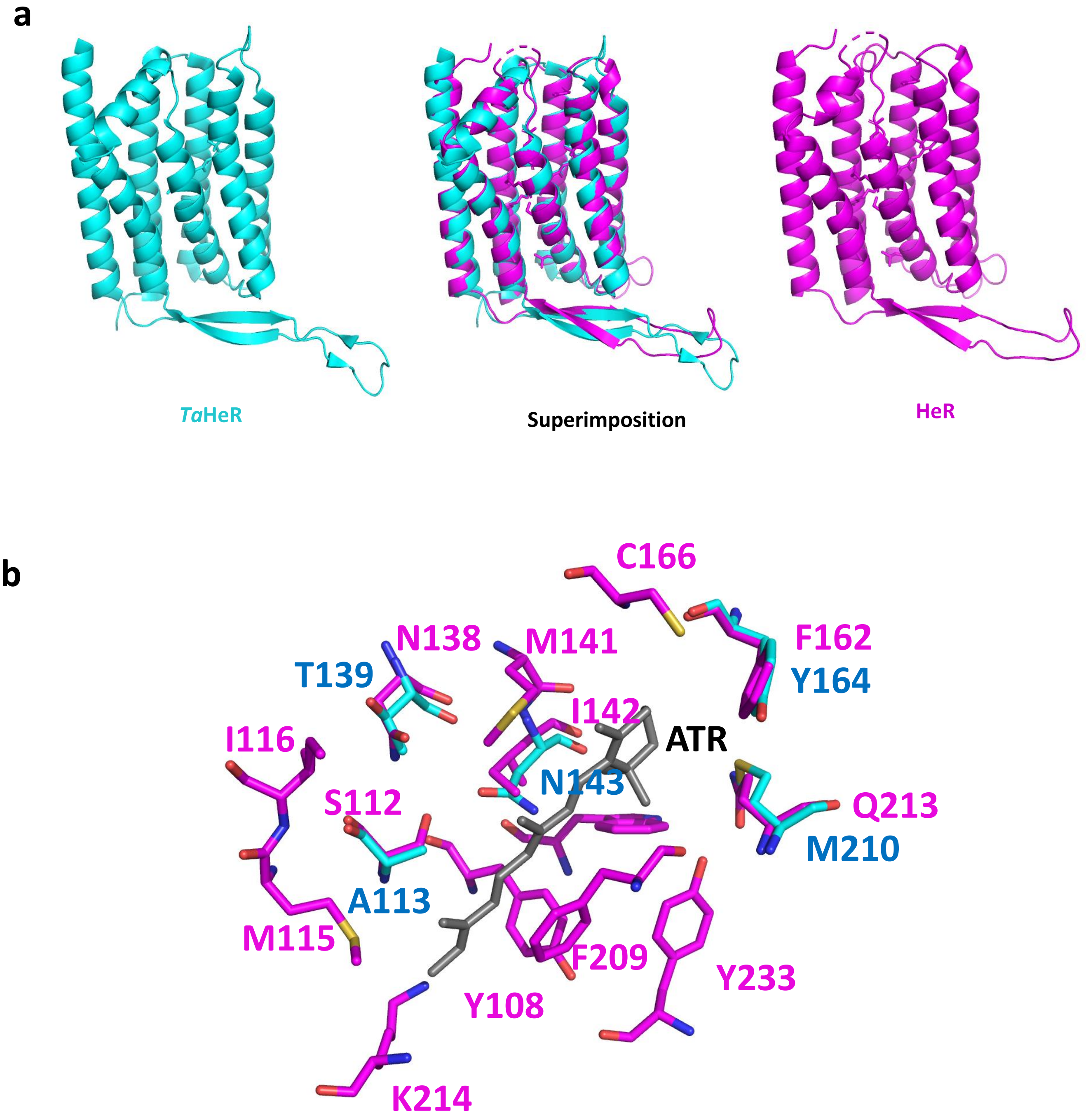


**Supplementary information, Fig. S9** A comparison of *Ta*HeR structure (6IS6) with HeR structure. **a**. Superposition of the two structures. The left is *Ta*HeR; the right is HeR 48C12; and the middle is the superimposition of the two structures**. b**. Comparison of the retinal binding pockets of the two HeR proteins. Residues colored in magenta are from HeR 48C12; and those in cyan are from *Ta*HeR. The retinal and residues of *Ta*HeR identical to those from HeR 48C12 are omitted for a clear view.
